# Editorial: Criticality in neural network behavior and its implications for computational processing in healthy and perturbed conditions

**DOI:** 10.3389/fncir.2022.1041250

**Published:** 2022-10-14

**Authors:** Ioanna Sandvig, Axel Sandvig

**Affiliations:** ^1^Department of Neuromedicine and Movement Science, Faculty of Medicine and Health Sciences, Norwegian University of Science and Technology (NTNU), Trondheim, Norway; ^2^Department of Pharmacology and Clinical Neuroscience, Faculty of Medicine, Umeå University, Umeå, Sweden; ^3^Department of Community Medicine and Rehabilitation, Faculty of Medicine, Umeå University, Umeå, Sweden

**Keywords:** neural systems, emergence, self-organization, critical state, neuronal avalanche

The aim of this Research Topic is to summarize the current state-of-the-art in the context of key conceptual, methodological, and analytical tools concerning criticality in neural networks within the context of the brain connectome. This includes emergent behavior such as memory and cognition, dynamic morphology-activity relationships at the micro, meso, and macroscale in response to perturbations, as for example, trauma or neurodegenerative disease. As such, the topic provides insights and perspectives on the relevance of criticality in the context of studying and understanding information processing in neural networks in health and disease in preclinical models and in the clinic.

Zimmern takes into consideration the clinical relevance of the topic and presents a comprehensive overview and explanation of central concepts and terminology in criticality, such as power laws, phase transitions, and the branching processes. The article provides a discussion and critique regarding the application of such concepts in the analysis of human neural data, with special focus on current controversies in the literature, and concludes with recommendations about how brain criticality may in the future add the diagnosis and treatment of diseases affecting the brain.

Carvalho et al. examine scaling relations observed in experimental data obtained from the anesthetized rodent cortex, which suggest a phase transition in firing rate variability that apparently differs from the canonical model of brain criticality and the branching process. The authors apply subsampling and two different models within the same universality class as the branching process and demonstrate that the experimental results can be reproduced in this manner.

Bellay et al. investigate how single neuron activity contributes to avalanches observed in the primate cortex by comparing LFP recordings obtained from multiple sites with concomitant single neuron extracellular and intracellular activity. Their results support a selective contribution of single neurons to specific LFP-based avalanche patterns and thus align with the notion that information processing in the cortex is supported by Hebbian cell assemblies.

Heiney et al. discuss the role of criticality in neural systems taking into consideration the principles of self-organization and neuroplasticity, within the context of the dynamic, reciprocal relationships between underlying network topologies and function. The review highlights the relevance and application of criticality for experimental neuroscientists, especially in terms of how changes in underlying structure-function relationships, for example due to damage or disease, affect critical dynamics and neural computation. As such, the article also provides insights as to the role of criticality for clinical translation.

Gross presents a dynamical systems perspective and its implications on criticality and neural dynamics. The article provides a comprehensive overview of bifurcation theory concluding that several critical manifolds, rather than one critical state, can explain neural dynamics, such as self-organization and information processing. The article thus highlights the need for the development of new theoretical models that take the high-dimensional parameter into consideration.

Shaheen et al. present a mathematical model that can be applied in the investigation of altered neuron-glial interactions as an underlying neuropathology, such as Alzheimer's disease. The model proposes a shift of astrocytic function toward exosome-dependent release of Ca^2+^ that might contribute to the accumulation of pathological protein aggregates.

Arvin et al. examine how underlying network topology is associated with neural activity and critical dynamics. By applying the small-world model of Watts and Strogatz and Kuramoto's model of coupled oscillators, the authors demonstrate that the dynamics of the system are shaped by short-range connections, while the state of the system, for example its response to a perturbation, is driven by long-range connections. This differential but synergistic contribution of short- and long-range connections thus confers the required neuromodulation to the system.

Alamian et al. assess baseline cognitive function in schizophrenia patients using magnetoencephalography and reveal changes in self-similarity and multifractality values in affected brain regions consistent with altered criticality properties, thus illustrating the relevance and potential application of criticality in the evaluation of this patient group.

Finally, Beggs addresses controversies in the field regarding the criticality hypothesis, which poses that healthy biological neural networks demonstrate optimal information capacity when they operate at the near critical point. The author concludes that such controversies are an essential element of scientific discourse and, as such, they are valuable for the refinement of relevant research questions in the field.

## Author contributions

Both authors listed have made a substantial, direct, and intellectual contribution to the work and approved it for publication.

## Conflict of interest

The authors declare that the research was conducted in the absence of any commercial or financial relationships that could be construed as a potential conflict of interest.

## Publisher's note

All claims expressed in this article are solely those of the authors and do not necessarily represent those of their affiliated organizations, or those of the publisher, the editors and the reviewers. Any product that may be evaluated in this article, or claim that may be made by its manufacturer, is not guaranteed or endorsed by the publisher.

